# The impact of COVID-19 related lockdown restrictions on musculoskeletal health: a systematic review

**DOI:** 10.1007/s00296-023-05406-2

**Published:** 2023-08-10

**Authors:** Tadesse Gebrye, Faatihah Niyi-Odumosu, Joyceline Lawoe, Chidozie Mbada, Francis Fatoye

**Affiliations:** 1 Department of Health Professions, Faculty of Health, Psychology and Social Care, Manchester Metropolitan University, Brooks Building, 53 Bonsall Street, Manchester, M15 6GX UK; 2 Centre for Health and Clinical Research, Faculty of Health and Applied Science, University of the West of England, Frenchay Campus, Coldharbour Lane, Bristol, UK; 3 Sunyani Technical University, Sunyani, Ghana; 4 Lifestyle Diseases, Faculty of Health Sciences, North-West University, Potchefstroom, South Africa

**Keywords:** COVID-19, Musculoskeletal, Lockdown, Systematic review

## Abstract

There is limited empirical evidence on whether Coronavirus disease 2019 (COVID-19) related lockdown restrictions precipitate or perpetuate musculoskeletal (MSK) disorders. This study was aimed to synthesis literature that assessed the impact of COVID-19 related lockdown restrictions on MSK health. A literature search was conducted using MEDLINE, CINAHL, PsycINFO, Allied and Complementary Medicine Database (AMED), Web of Science, and Scopus databases. Studies meeting the following criteria were included in the review: the condition being considered was MSK health, the intervention was COVID-19 related lockdown restrictions, cross sectional studies, cohort studies, case controlled, prospective studies and retrospective studies. Data were extracted by 2 independent researchers. Risk of bias was assessed by the Newcastle–Ottawa quality assessment scale. Evidence from included studies was summarised using narrative synthesis. Fourteen studies comprising 22,471 participants of the general population from Turkey (n = 5), Italy (n = 1), Poland (n = 1), Australia (n = 2), Jordan (n = 1), Bangladesh (n = 1), Estonia (n = 1), the Netherlands (n = 1) and Saudi Arabia (n = 1) have met the inclusion criteria. The sample size of populations studied ranged from 91 to 1054. The included studies used questionnaire, visual analogic scale, or growth mixture modelling. Except for one study, all the included studies reported increased prevalence and incidence of MSK disorders due to COVID-19 related lockdown restrictions. The findings suggest that COVID-19 related lockdown restriction led to increased MSK disorders. Home-based strategies such as physical activity programmes and ergonomic workspace could potentially guide public health authorities to avoid MSK health problem.

## Introduction

In December 2019, SARS-CoV-2 or Coronavirus disease 2019 (COVID-19) emerged as a new disease in China [1], then World Health Organisation (WHO) recognised it as a pandemic on 11 March 2020 [2]. Infection with COVID-19 can present asymptomatically or with severe or critical illnesses affecting primarily, the cardiopulmonary systems [3]. In early of August 2020, 18 million and over 690,000 people were affected and died by COVID-19 worldwide [4]. Between February and November 30, 2020, 1,651,229 positive cases of COVID-19 were diagnosed, and 57,647 died in Italy [5]. In addition to the mortality resulting from COVID-19, it creates a threat of indirect morbidity resulting from other preventable diseases that could have managed but for disruption of essential health services [6].

In an attempt to curtail disease spread, COVID-19 related lockdown restrictions and other restriction measures were imposed across the globe [7–9]. However, conceptualisation and implementation of lockdown restrictions differed across different countries [7, 9, 10]. Essentially common to most restriction measures were stay-at-home orders or other equivalent interventions involving restriction of movement and social contacts [7, 9, 10]. Emerging data suggests that the health impact of lockdowns during COVID-19 may be comparable or worse than the pandemic itself in most situations [9]. However, weighing up the ultimate costs and benefits of lockdown measures is a challenge [8], as it is difficult to ascertain whether lockdowns have directly caused the negative health hazards or whether the hazards are a direct effect of the inherent health disaster of the pandemic [9]. Haileamlak, [6] stated that COVID-19 related lockdown restrictions impacted health systems significantly, and in some instances led to limited availability and utilisation of services. Similarly, the socio-economic and behavioural impact of COVID-19 lockdowns on the general population include business closures, and transition to working from home [11, 12]. People working in different sectors had to adapt to working from home during the COVID-19 pandemic, the impact of this transition on musculoskeletal (MSK) health was unknown [13].

Emerging literature suggests that COVID-19 has direct effects on the MSK system, commonly presenting as myalgias, arthralgias, and neuropathies/myopathies [14]. Also, in most reports of long COVID, MSK pain prevalence of between 25 and 50% have been reported [15]. However, there is limited data on the effect of COVID-19 related lockdown restrictions on incidences and prevalence of MSK disorders. There are social, behavioural and health challenges that come with working from home including life and work balance, need to set up a proper workplace at home, caregiving responsibilities, mental well-being, and risk of obesity [16]. Furthermore, known patients with MSK disorders could experience relapse or exacerbation of their conditions, as well as face delays in timely access to the hospital as a result of fear of exposure to the infection, or COVID-19 related movement restrictions itself [17]. Therefore, lockdown restrictions provide a breeding ground for more MSK disorders, as well as a high risk of neglecting individuals with MSK disorders even when their need is greatest, while much attention is devoted to curbing transmission of infection and save patients’ lives. To date, no literature review has been published summarising the impact of COVID-19 related lockdown restrictions on MSK health. The aim of this study was to summarise the literature that assessed the impact of COVID-19 related lockdown restrictions on MSK health.

## Methods

We searched for published article that assessed the impact of COVID-19 related lockdown restrictions on MSK health. This study was performed and reported following the Preferred Reporting Items for Systematic Reviews and Meta-Analyses checklist for systematic reviews of intervention [18]. A protocol for this systematic review was prospectively registered on PROSPERO and can be found at https://www.crd.york.ac.uk/PROSPERO/display_CRD:42,022,307,074) .

### Data sources and search strategy

On March 5, 2023, we performed a comprehensive search of the following databases: MEDLINE, CINAHL, PsycINFO, Web of Science, and Scopus, and Allied and Complementary Medicine Database (AMED). A further updated search until July 17, 2023 were also performed. The searches were combinations of COVID-19 or coronavirus or 2019-ncov or sars-cov-2 or COVID-19; musculoskeletal disorders, and musculoskeletal problems (Appendix 1). The search was delimited to articles published in English language. References of the included studies was performed for any studies we missed during the electronic search.

### Inclusion and exclusion criteria

Studies were considered eligible for inclusion if they fulfilled the following criteria: the condition being considered was MSK disorders (> 18 years of age, no restriction of sex, and race), and the intervention was COVID-19 related lockdown restrictions. The definition of MSK disorders include injuries or pain in the human MSK system such as the joints, ligaments, muscles, nerves, tendons, and structures that support limbs, neck, and back. We included cross sectional studies, cohort studies, case controlled, prospective studies and retrospective studies, and the background of study is COVID-19 related lockdown restrictions. We excluded studies that had reviews, editorials, conference papers, case report or series study, and animal experiments. Two reviewers (TG & CEM) independently screened the search results using the criteria mentioned above. When the judgments of both reviewers were not similar, other reviewers solved the discrepancy (FF).

### Study selection and assessment of methodological quality

Following removal of duplicates, one reviewer (TG) screened all titles, abstracts, and full-text articles and a sample of each was checked by a second reviewer (CEM). Any difference was resolved by discussion and consensus with the third reviewer (FF). Full texts of the identified studies were checked against the inclusion and exclusion criteria. The quality assessment of the risk of bias of the included studies were evaluated using the Newcastle–Ottawa quality assessment scale [19]. Studies were scored using a scale with a possible maximum of nine points where a score ≥ 7 indicated low, a score between 5 and 6 as moderate and a score ≤ 5 stars as high risk of bias with an overall quality score of 9 stars.

### Data extraction

Excel sheet was used to extract data for the prespecified outcomes including author, country, study design, sample size, outcome, outcome measure and results or key findings. Data extraction and determination of information eligibility were conducted by two reviewers (TG & CEM) independently following the criteria above, while discrepancies were resolved by consensus or with a third reviewer (FF), as appropriate.

### Data synthesis

Study data were extracted by three reviewers (TG, CEM & FF) into a template. Findings for each study focusing on the impact of COVID-19 related lockdown restrictions on MSK disorders data were then summarised by one reviewer (TG), and the summaries discussed and modified by the research team as necessary, to generate an overall conclusion about the association of COVID-19 related lockdown restrictions and MSK disorders.

## Results

**Fig. 1:**
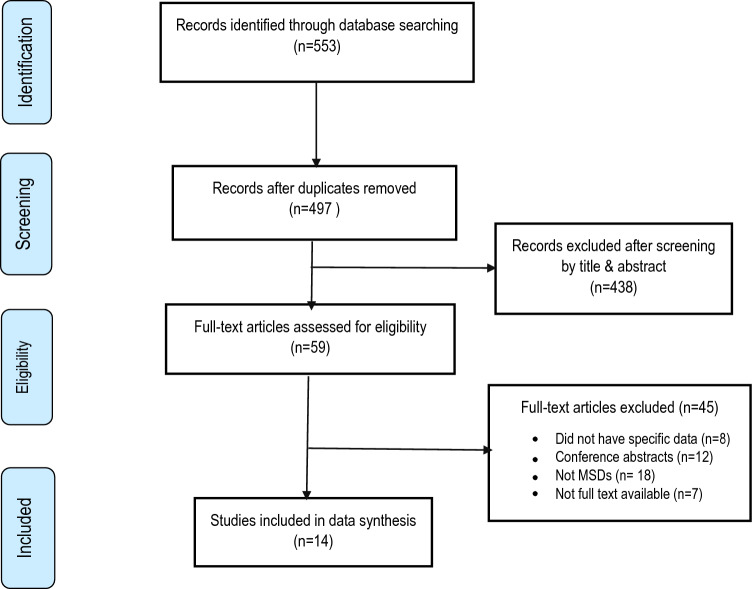
Flow diagram of publications included and excluded in the review

The searches generated 535 (284 records in MEDLINE, EMBASE, AMED, CINAHL, and PsycINFO, 156 records in Web of Science, 95 records in Scopus) studies. A total of 56 articles were duplicates. After reviewing their titles and abstracts, 438 studies were excluded. A total of 41 potentially relevant records were retrieved for detailed full-text evaluation. Finally, 14 articles met the selection criteria and were deemed to contain data relevant to the systematic review and were included. A further updated search yield no new articles. The flowchart detailing the results of the literature selection process is shown in Fig. 1.

### Study population and sample size

Studies varied in both characteristics of populations studied and sample size (Table 1). The included studies included a wide range of demographic characteristics that were published between 2020 and 2022. The included studies were conducted in Turkey [20–24], Italy [25], Polland [26], Australia [13, 27], Jordan [28], Bangladesh [29], Estonia [30], the Netherlands [31], and Saudi Arabia [32]. The sample size of populations studied ranged from 91 to 40,702 participants.

**Table 1:** Characteristics of studies included in systematic review reporting on the impact of lockdown on the general population

Authors/country	Study design	Population	Outcome	Outcome measures	Key findings	Comments
Grabara and Sadowska-Krępa [26], /Polland	A cross-sectional design	373	MSK pain intensity	Standardised NMQ	#Prevalence of LBP (36%), knee pain (22.5%y), and neck pain 21%	The highest average pain intensity was reported for the low back and knees
Yorulmaz et al. [20] /Turkey	Cross-sectional study	381	MSK system pain intensity	The Work Environment Evaluation Questionnaire, and the MSK Pain Intensity Assessment Questionnaire	The rate of academicians with MSDs increased to 70.6%	Disorders related to the eye, neck, back, elbow, hand–wrist, thigh, knee, and foot–ankle increased
Ahmed et al. [29] /Bangladesh	A cross sectional study	230	#Assessment of MSK disorder or pain	Numeric pain rating scale and Nordic MSK Questionnaire	Significant difference observed (p < 0.0001) in pain intensity	Lockdown has negatively impacted the MSK health of the participants
Arca et al. [23] /Turkey	Cross-sectional study	158	Individual's pain	#The Nordic MSK questionnaire #Visual analog scale (VAS)	Neck pain presence was 73.4%	Neck and back pain was the most common
Bosma et al. [31]/The Netherlands	A cohort study	40,702	KSK pain Working situation	Questionnaire	Working from home was associated with higher risks of having MSK pain in the lower back, upper back, neck, shoulder and/or arm	Home workers had higher risks of having MS pain
Snodgrass et al. [13] /Australia, United Kingdom, United States	Online survey	511	Prevalence, intensity and impact of MSD	#Nordic questionnaire modified to include head pain #26 with validated questionnaires to quantify physical disability related to a respondent’s specific symptoms	#89% reported some MSK pain #Work location was associated with upper back pain (p = 0.011); body posture with headache (p = 0.027) and LBP (p = 0.003)	Nonergonomic work environment so frequent computer users during COVID-19 are related to having upper back pain, whereas nonergonomic postures are related to having headache and LBP
Toprak et al. [21]/Turkey	Case-controlled study	686	MSK pain	#The Turkish version of the NMQ #Covid-19 Phobia Scale (C19P-S) #The Turkish version of the Jenkins Sleep Scale (JSS-T)	#LBP was higher in the stayed home group #Neck pain, upper back pain, shoulder pain, and elbow pain decreased #Wrist/hand pain, hip/thigh pain, knee pain, and ankle/feet pain unchanged	Individuals who stayed home had more MSK complaints than those who continued work during the Covid-19 lockdown
Oakman et al. [27] /Australia	Survey	488	MSK discomfort	GMM	High stable (36.5%), mid-decrease (29.7%), low stable (22.3%) and rapid increase (11.5%) MSK pain	Employers need to optimise working conditions to reduce MSK pain in employees working from home
Dolci et al. [25] /Italy	A cohort study	17,591	Epidemiology of fractures and MSK traumas	Emergency departments (ED) databases and trauma registries from 3 Trauma centres	ED trauma visits decreased by − 59.8%	Observed an increased proportion of traumas
Argus and Pääsuke [30] /Estonia	Online questionnaire	161	#MSK complaints #Self-reported physical activity (PA)	#Baecke Physical Activity Questionnaire, #NORDIC MSK Questionnaire	#No significant differences in the prevalence of MSK pain #A significant reduction in total PA #A significant increase in work-related PA	No change of prevalence of MSK pain
Alzeyadi et al. [32]/Saudi Arabia	Cross-sectional design	353	Prevalence of MSK disorders	Online questionnaire	#Change their work shift due to COVID-19 (71.6%); not due to COVID-19 (60%) and those who didn't change their work shift at all (27.7%)	Change due to COVID-19 duty had comparatively more MSDs than others
Şengül et al. [24]/Turkey	Descriptive design	1138	MSK System discomforts	Cornell MSK Discomfort Questionnaire	A statistically significant difference of the pain level (p < 0.001)	An increase in the severity of the emergent discomforts during COVID-19
Salameh et al. [28]/Jordan	Cross-sectional descriptive study	91	The presence or absence of MSK complaints	NMQ plus body map, Persian version of work ability index (WAI) questionnaire, and Health and Safety Executive (HSE)	#96.7% reported at least one MSD #The most common region with pain and discomfort was neck	A prolonged period of emergency distance learning induced MSK discomfort in the form of pains
Is et al. [22]/Turkey	Google Forms web survey platform	310	MSKl pain and its relationship with physical activity status	MSK questionnaire	#There was a statistically significant difference (p < 0.001) between physical activity habits #No statistically significant difference between the duration of physical activity and the MSK pain	Pandemic caused an increase in MSK pain

Note: *MSD* musculoskeletal disorder; *LBP* low back pain; *NMQ* nordic musculoskeletal questionnaire; *MSK* musculoskeletal

Note: *GMM* growth mixture modelling**;** *NMQ* nordic musculoskeletal questionnaire; *MSK* musculoskeletal; *MSD* musculoskeletal disorder

### Study design

All included studies were cross-sectional in nature and did not follow participants over time. Five studies used survey method [13, 20, 22, 27, 30, 31], while eight studies were based on descriptive cross-sectional design [20, 23, 24, 26, 28, 29, 32]. The remaining study was case controlled [21].

### Measurement of health outcomes

Studies used a variety of instruments to assess the impact of COVID-19 related restrictions lockdown on MSK disorders. Eight studies used Nordic MSK Questionnaire [13, 23, 26, 28, 29, 29–31]. One study used Visual Analogic scale [23]. The Work Environment Evaluation Questionnaire was used by Yorulmaz et al. [20]. Three studies [22, 24, 32] used questionnaire whereas Oakman et al. [27] utilised Growth Mixture Modelling.

### Study quality assessment and risk of bias

The quality scores of the studies can be found in Table 2. After assessing the study quality by the Newcastle Ottawa scale, five studies [13, 23, 26, 27, 30, 31] received a quality score of 8, five studies [20, 22, 24, 28, 32] received a quality score of 7, and the remaining two studies [21, 29] received a quality score of 6.

**Table 2:** Quality assessment of the included studies

Study	Selection 1*	Selection 2*	Selection 3*	Selection 4*	Comparability 1**	Exposure 1*	Exposure 2*	Exposure 3*	Total (Max. 9)
Yorulmaz et al. [20]	1	1	1	1	1	1	1	0	7
Grabara and Sadowska-Krępa [26]	1	1	1	1	2	1	1	0	8
Toprak et al. [21]	1	1	1	1	1	1	0	0	6
Snodgrass et al. [13]	1	1	1	1	1	1	1	1	8
Bosma et al. [31]	1	1	1	1	1	1	1	1	8
Oakman et al. [27]	1	1	1	1	2	1	1	0	8
Salameh et al. [28]	1	1	1	1	1	1	1	0	7
Dolci et al. [25]	1	1	1	1	1	1	1	0	7
Is et al. [22]	1	1	1	1	1	1	1	0	7
Ahmed et al. [29]	1	1	1	1	1	1	0	0	6
Arca et al. [23]	1	1	1	1	2	1	1	0	8
Argus and Pääsuke [30]	1	1	1	1	1	1	1	1	8
Alzeyadi et al. [32]	1	1	1	1	1	1	1	0	7
Şengül et al. [24]	1	1	1	1	1	1	1	0	7

### Overall study results

Assessed by the Nordic MSK Questionnaire, the impact of COVID-19 related restrictions lockdown on MSK health increased during COVID-19 related lockdown restrictions [13, 21, 26, 28–31]. Disorders related to the eye, neck, back, elbow, hand–wrist, thigh, knee, and foot–ankle increased [20]. For example, the 12-month prevalence of low back pain, knee pain, and neck pain on army soldiers was reported as 36%, 22.5% and 22.5%, respectively [26]. A pandemic-related general worsening of the chronic pain experience with a more detrimental impact to women relative to men was reported in Turkey due to COVID-19 related restrictions lockdown [24]. Contrary to the findings of the included studies, one study that assessed the consequences of COVID -19 related lockdown restrictions on MSK disorders reported that lockdowns did not change the prevalence and incidence of MSK disorders among office workers and professional soccer players [30].

## Discussion

Lockdown restrictions imposed by COVID-19 predispose to an increased risk of MSK disorders due to working from and at non-ergonomic home environments, sedentariness, coupled with limited access to healthcare facility for chronic conditions even when the need was greatest. This is the first systematic review to assess the impact of COVID-19 related lockdown restrictions on MSK health. Importantly, the included studies were methodologically of good quality. Moreover, the majority of studies reported risk of bias in one or more of the categories used to assess risk of bias. Except for one study [30], all the included studies reported that COVID-19 related lockdown restrictions had a negative impact on MSK health affecting several regions of the body including lower back, neck, wrist, hip, ankle and shoulder. A significant increase in pain was observed among the general population during the lockdown periods.

No association were observed between COVID-19 related lockdown restrictions and MSK health among office workers in Estonia [30]. The major reasons suggested for this finding include maintaining of habitual physical activity level and preparing a more comfortable and ergonomic workspace by office workers [30]. However, the authors highlighted that on an individual level, a larger decrease in sport-related physical activity can be associated with MSK pain in several body regions.

Evidence from the current review demonstrates that more females suffered from MSK disorders during the COVID-19 related lockdowns than their male counterparts as their pain intensity levels were higher than those of males [20, 24]. Other authors also stated that females suffered from neck, shoulder, and back pain more than males and the loss of productivity due to MSK health is higher than in males during the pandemic [33, 34]. The main reasons for more females suffering from MSK disorders may include females having less muscle tissue and more adipose tissue, conducive to pregnancy, and have different physiology compared to males [33, 35]. The sex difference pattern in COVID-19 related MSK disorders observed in this review is consistent with earlier submissions. Specifically, Hart [36] suggests that the higher risk for injuries to tissues of the MSK system in females is attributable to hormonal changes across lifespan, but markedly at onsets of puberty and menopause; gene expression independent of sex hormones; and immune dysfunction. In line with the foregoing, Wolf et al. [37] conclude that structural anatomy differences, hormones, and genetics are important considerations in sex difference in MSK disorders between males and females.

Prior to the COVID-19 pandemic, the burden of MSK condition was predicted to escalate as the global population age and the prevalence of risk factors increases [38–40]. Emerging evidence suggests that the advent of COVID-19 seemed to further exacerbate the already increasing MSK health crisis. Thus, containment approaches such as self-isolation and limited activities, promote sedentariness and chronicity of already present MSK conditions.

There are certain strengths and limitations to the current study. In this review, we used a systematic approach such as the screening of numerous data bases, the involvement of multiple reviewers, and assessment of methodological quality of the studies. Only English language studies were included. Therefore, it is possible that relevant literature published in other languages may have been excluded. Also, the included studies in the present review were cross-sectional in nature, therefore, it is difficulty to make a firm conclusion on the causality between COVID-19 lockdown restrictions on MSK health, as the findings of those studies may not be true reflection of the population. It was not possible to undertake a meta-analysis for the included studies due to the adoption of different outcomes and did not present mean or standard deviation (SD) for the outcomes [41]. Further, we found one study precluding any meaningful conclusions on the consequences of COVID-19 related lockdown restrictions on MSK health [30]. However, the review highlights that there were MSK complaints during the Covid-19 related lockdowns. Therefore, to prevent the consequences of COVID-19 related lockdown restrictions MSK health, it is important that home-based strategies such as physical activity programme and ergonomic workspace should guide public health authorities to reduce the burden MSK disorders.

## In conclusion

 The findings of the review indicated that COVID-19 related lockdown restrictions negatively impacted on MSK health, affecting several regions of the body including lower back, neck, wrist, hip, ankle and shoulder. It also has a prolonged impact upon daily life for a large proportion of the general population. Understanding the exact impact of COVID -19 related lockdown restrictions on MSK health is of great importance to better prepare for potential future waves of this or other pandemic. Thus, the current results provide insights for health professionals and policymakers to put in place appropriate preventive measures during public health emergencies such as COVID-19 to reduce their burden on musculoskeletal health.

## Data Availability

All data related to this work are available in this research article.

## Appendix 1 Search strategy

S11 S9 AND S10

S10 S6 OR S7 OR S8

S9 S1 OR S2 OR S3 OR S4 OR S5

S8 AB musculoskeletal injurie

S7 AB musculoskeletal pain

S6 AB musculoskeletal disorders

S5 AB cov-19

S4 AB sars-cov-2

S3 AB 2019-ncov

S2 AB covid-19 or

S1 AB coronavirus
